# The Higher the Children's Achievements, the Better the Elderly Health? Evidence From China

**DOI:** 10.3389/fpubh.2022.871266

**Published:** 2022-05-26

**Authors:** Pei ru Zhang, Yiwei Liu

**Affiliations:** ^1^School of Marxism, Anyang Institute of Technology, Anyang, China; ^2^School of Government, Central University of Finance and Economics, Beijing, China

**Keywords:** children's achievement, elderly health, housing arrangements, economic contribution, social capital

## Abstract

Health, an important indicator for measuring the elderly's life and wellbeing, is an important part of positive and healthy aging. Children's achievements are closely linked to their parents' health. However, existing literature does not cover how children's achievements impact the health of their elderly parents. Data were derived from the 2014 Chinese Longitudinal Aging Social Survey; this study includes 6,793 elderly people ages 60 and older as samples. A multiple linear regression model was used to analyze the correlation between children's achievements and their elderly parents' health statuses in China. The results show that the higher the children's income and education, the better their health of their elderly parents. Living patterns, children' financial support to their parents, and social capital play a mediating role in the relationship between children and their elderly parents. These findings provide further insight into potential factors associated with the children's achievements and elderly health.

## Background

According to United Nations standards, in 1999, China entered an aging society and has experienced a gradual increase in the aging population since. In 2020, there are 260 million people ages 60 and older, which accounts for 20.3% of the total population. Out of 260 million people, 168 million are ages 65 and older, which accounts for 12.2% of the total population. According to the World Health Organization's forecast, over 30% of China's population will be over 60 years old by 2050, and China will be the fastest aging country in the world. The Chinese society will face irreversible aging during the twenty-first century, which will impose a major challenge to China (1). Due to its profound intrinsic value, elderly health often serves as an important dimension for evaluating social development in an aging society. However, the overall health of the elderly population deteriorates due to factors such as physiological aging, and various health problems appear over time (2). Therefore, the improvement of health within the elderly population and reduction in incidence rate reduces pain associated with diseases and helps improve elderly individuals' happiness and quality of life.

Many studies have analyzed the influencing factors of the health of the elderly, and the main viewpoints can be summarized into the following two aspects: on the one hand, objective social resources owned by the elderly, including medical insurance, pension (3). On the other hand, social participation of the elderly, including intergenerational care and voluntary service (3). In addition, some studies discussed the relationship between children's marriage, children' financial support to their parents and health of the elderly based on the perspective of intergenerational relationship. However, there is no study analyzed the impact of children's achievement on the health of older persons. In fact, among the many factors influencing elderly health, children's achievements (mainly educational level and income status) play an important role (4).

In the traditional Chinese culture of filial piety, the concept of raising children to care for their elderly family members still dominates society. With higher achievements, children can provide more children' financial support to their parents and psychological satisfaction (Chinese have the idea of “face”), which affects their aging parents' health (5). However, another factor is that higher achieving children are more likely to seek careers development away from their hometowns. Children from rural areas are more likely to settle in large cities away from their families. This lowers children's spiritual consolation toward the elderly and reduces children's daily care of the elderly, which in turn affects their aging parents' health status (3). It is indicated that no consensus has been reached on the impact of children's achievements on the health of elderly parents. For China, scientific methods and data are needed to explore the impact of children's achievements on the health of elderly parents in China, which has the largest elderly population.

This study attempts to use Chinese data to investigate how children's achievements impact the health of their elderly parents within China's increasingly aging population. This article makes three contributions to the existing literature. First, this study contributes to the growing literature on the relationship between children's achievements and elderly health. Second, to the best of our knowledge, it provides the first evidence of relationship between children's achievements and elderly health. Third, we investigate how obesity is associated with happiness through children' financial support to their parents, living patterns, and social networks. The findings may also be applicable beyond China.

## Literature and Research Hypotheses

### Impact of Children's Achievements on Elderly Health

Currently, there are relatively few studies on the relationship between children's achievements and their elderly parents' health. Children's achievements not only include multiple dimensions, such as income and education level. However, only a few studies have analyzed the relationship between children's education levels and their elderly parents' health (6). For example, Zimmer et al. (4) used data from the 1989 survey on elderly health and living status in Taiwan and found that children's education levels are significantly correlated with their parents' risk of death. Compared to children with junior high school education level and below, children with college degrees can reduce the risk of parental death by 20%. A Swedish study also confirmed that children with college degrees can reduce the risk of their parents' death by ~20% compared to children with compulsory education (7). A study in Mexico found that children's education did not significantly improve their parents' short-term physical function but could reduce long-term parent mortality. Compared to families in which none of the children received college educations, families in which all children received college degrees, fathers were 25% less likely and mothers were 29% less likely to die (8). Lei et al. (9) studied the impact of spousal and child education on the mortality of in the elderly and found that the education levels of individuals, children, and spouses can significantly reduce one's mortality. Compared to families in which children only received an elementary level education and lower, families in which children received a high school education or higher were 15% less likely to face mortality. From this perspective, we propose the following hypotheses:

*Hypothesis 1: The higher the children's achievements, the better the health of their elderly parents*.

### The Construction of Mechanisms by Which Children's Achievements Affect the Health of the Elderly

#### Living Patterns

In traditional Chinese society, “filial piety culture” emphasizes that children cohabit with their parents in order to take care of their aging parents; however, children with higher achievements have greater autonomy and say in terms of living style (10). Miron (11) studied from the perspective of housing affordability and found that the income level of children has an important impact on the living arrangement of the elderly. Wang et al. (12) used Chinese urban data to carry out research, and the results also showed that education level and income level were positively significant with the intergenerational separation rate. If the children have a high enough income and education level, there is no reason to cohabit with the elderly for economies of scale (13).

Intergenerational cohabitation can significantly improve the health of the elderly (14). On the one hand, living with children brings both financial and material support and psychological satisfaction to avoid poverty, social isolation and declining mental health of older people (15). On the other hand, Moritz and Satariano (16) showed that although elderly people who live alone are less dependent on other people's functions in their living arrangements and make greater efforts to monitor their own health to avoid serious medical problems, as the health status of the elderly deteriorates, they often move in with their children or others (17). Based on this, this study proposes the following research hypothesis.

*Hypothesis 2: Living patterns play a mediating role in the relationship between children's achievements and their elderly parents' health*.

#### Children' Financial Support to Their Parents

It is a traditional Chinese concept “bring up children for one's old age.” The cultural norms of the family clan confine children to care for the elderly. Liu et al. (2) found that the level of children's achievements determines the “opportunity cost” in providing life care for the elderly. Children with relatively low achievements assume the role of “primary caregiver,” while children with higher accomplishments assume the responsibility of elderly care through economic compensation. Silverstein et al. (18) found that an increase in children's wealth will increase their children' financial support to their parents of the elderly. Although children and the elderly may live separately, the close emotional connection brings wealth effects and economic transfer; children are also more likely to provide financial assistance and material transfer to the elderly (19).

With the deepening trend of socialization in family functions, children have become the main providers of long-term health and support for the elderly. In most cases, they constitute the pillar of their support system and protect their health through the transfer of economic resources. Studies have shown that elderly individuals with more economic resources have better health (20). On the one hand, in Chinese culture, filial piety is a factor that accounts for parental support expectations. The elderly expects their children to be their main supporters. Fulfillment of this intergenerational support expectation can effectively improve mental health in the elderly (21). On the other hand, due to the incomplete social security system in China and insufficient coverage of the formal social support system, children's economic contribution brings medical and assistance advantages to the elderly in China. These economic resources can be converted into nutrition, medical treatment, and possible leisure time, which will result in a higher living standard and corresponding health improvement for the elderly (22). Based on this, this study proposes the following hypotheses:

*Hypothesis 3: Children' financial support to their parents plays a mediating role in the relationship between children's achievements and their elderly parents' health*.

#### Social Capital

In traditional Chinese society, children's achievements directly affect the social capital of elderly parents. Studies have found that the higher the achievement of their children is, the richer the social capital of elderly parents (19). In other words, when children's academic performance or professional status is higher, relatives and friends may strengthen the connection with the elderly with higher achievement of children, so child achievement can improve the social capital accumulation of elderly parents (23) found that the children's socioeconomic status directly affects the social capital of the elderly. If the children's socioeconomic status is higher, the elderly's relatives, neighbors and friends interact with the elderly more frequently.

Studies have shown that social capital plays a positive role in promoting the health of the elderly (24). Baker and Silverstein (25) proposed that social capital increases the possibility of the elderly's participation in social activities. In social activities, the elderly's needs for life, leisure, and fitness activities are met, and the sense of belonging and accomplishment is enhanced and has a positive impact on the emotional health of the elderly. Pearlin et al. (26) found that rich social capital can maintain the elderly's sense of controllability in adversity and mediate their mental health. In addition, Cohen and Syme (27) found that rich social capital links the elderly to more social resources so they can better address possible negative situations in the future. Therefore, social networks play an important protective role in the transition from functional limitations to disability (28). From this perspective, we propose the following hypotheses:

*Hypothesis 4: Social capital plays a mediating role in the relationship between children's achievements and their elderly parents' health*.

## Research Methods

### Data Source

The data were derived from the 2014 Chinese Longitudinal Aging Social Survey. The Chinese Longitudinal Aging Social Survey (CLASS) is a nationwide and continuous large-scale social survey project designed by the Institute of Gerontology of Renmin University of China and was implemented by the China Survey and Data Center of Renmin University of China. The CLASS survey covers 28 provinces (cities and autonomous regions), except Hong Kong, Taiwan, Macau, Hainan, Xinjiang, and Tibet. The survey contents were expanded based on conventional survey items to focus on the mental health, social function, social network, and retirement planning for the elderly. It investigates the personal information of the elderly and focuses on the collection of information about the elderly's families and communities. This survey enables a comprehensive understanding of elderly individuals and offers information support for in-depth research on aging issues and the government's formulation of aging policies.

The survey adopted a stratified multistage probability sampling method. It selects county-level regions (including counties, county-level cities, and districts) as the primary sampling unit, village/residential committees as the secondary sampling unit, and draws sample households from each village/residential committee by image sampling. One elderly person was interviewed from each household. The survey subjects were the elderly individuals ages 60 and older and the staff of the survey community (village). Finally, the 2014 CLASS baseline survey samples came from 134 counties and districts and 462 communities across the country. Each community had an average of 25 samples. A total of 462 community survey questionnaires were collected, including 11,511 valid individual questionnaires for the elderly ages 60 and older. There are 558 elderly people with lack of health variables, 294 elderly people without children, 92 children with lack of educational information, and 107 elderly people with lack of demographic characteristics. Therefore, after excluding some missing variables and samples of the elderly without children, 10,460 elderly people were included in the analysis.

### Variable Selection and Description

#### Dependent Variable

The dependent variable in this study was the self-rated health, physical, and mental health of the elderly. Our study used multiple indicators instead of unit-dimensional standard measurement to measure health level. In particular, we used indicators of self-rated health (SRH), physical health utilizing the activities of daily living scale (ADL) and the instrumental activities of daily living scale (IADL), and mental health, which combined subjective and objective health assessment to more accurately measure the health of the elderly.

SRH has proven to be a relatively stable indicator for measuring health, and can effectively predict the risk of death for the elderly. Even when the objective health status is considered, self-assessed health remains a better representation of the health status of the elderly (2). The SRH measurement is based on the response to the question, “What do you think of your current physical health?”. The score is from 1 to 5. A higher score indicates batter health status.

For IADL items, the respondent was asked, “Do you have any difficulty in doing the following activities?” The activities include cooking, washing clothes, cleaning, taking medicine, nailing, managing money, making phone calls, getting out in the rain, shopping, and going to a physician. Based on these ten items of IADL, we constructed a dichotomous indicator for IADL disability, similar to previous similar studies of disability trends (29). There are three options for each question about instrumental ability of daily living (3 = need no help from others, 2 = need some help from others, 1 = completely need help from others). We summed up the scores for each item (10–30 points). A higher score indicates that the elderly had a stronger instrumental ability of daily living.

Depression is the most important indicator of mental health, which is often used to measure mental health status. People suffering from depression tend to have sub-health or even serious psychological problems. In this study, we used depression as a measure of the elderly's mental health. Center for Epidemiologic Studies Depression Scale-revised 10-item version for adolescents (CESDR-10) was used to measure the degree of depressive symptoms based on ten questions with three response options, “3 = no, 2 = sometimes, 1 = often.” Negative emotional responses were reverse scored to provide a positive score with a summed range of 10–30. Higher scores suggest a lower degree of depression and, conversely, better mental health.

#### Independent Variable

Achievement refers to an individual's attainment of his or her social identities. In the Chinese context, children are the hope and sustenance for parents who generally “cherish great ambitions for their son and daughter.” On the one hand, parents hope their children live better in the future; on the other hand, parents of promising children will be respected by others (2). The social achievements of children are mainly reflected in two aspects: education and income (30). To this end, this study considers children's education and income levels as proxy variables of children's achievement. In the CLASS questionnaire, the question regarding children's education levels is “What about your child's education level?” We converted it into continuous variables: 0 = illiterate, 6 = primary school, 9 = junior high school, 12 = high school, 15 = junior college, 16 = university, and 19 = master. The question regarding children's income level is “What about your child's financial situation?” We also convert it into continuous variables: 1 = very difficult, 2 = relatively difficult, 3 = general, 4 = relatively rich, and 5 = very rich. Since the elderly may have multiple children, the highest achievement among multiple children is usually taken to measure children's education and income levels.

#### Mediating Variable

The elderly can maintain their health through resources such as financial assistance, information, and emotional support (31). This study sets three variables: housing arrangements, children' financial support to their parents, and social capital.

In terms of housing arrangements, the study adopts the question “Who lives under the same roof and eats at the same table as you?” in the CLASS questionnaire. For the four options “son, daughter, son-in-law, daughter-in-law” one is assigned if at least one option is met; otherwise, zero is assigned.

The question regarding children' financial support to their parents is “In the past 12 months, did this child give you (or living spouse who resides with you) money, food or gifts? How much are these items worth?” Logarithm was used for the analysis.

In terms of social capital, the Social Network Scale (Lubben Social Network Scale 6, LSNS-6) compiled by Lubben et al. (32) in the CLASS questionnaire was used to measure the social capital of the elderly. This scale includes family and friend networks, which inquiries about the relatives and friends of elderly individuals who can meet, talk, and provide help. “None,” “1,” “2,” “3–4,” “5–8,” “9” and “more” are assigned with values 0, 1, 2, 3, 4, and 5, respectively. Therefore, the social network range of the elderly is between 0 and 30; the higher the score, the broader the social network of the elderly.

#### Control Variable

Based on the CLASS survey, this study selected the characteristic variables of the elderly as the control variables, mainly including elderly gender (dummy variable: 0 = female, 1 = male), age (continuous variable), marriage status (dummy variable: 0 = unmarried, divorced or widowed, 1 = has spouse), education level (continuous variable: 0 = illiterate, 6 = elementary school, 9 = junior high school, 12 = high school, 16 = university), place of residence (dummy variable: 0 = rural, 1 = urban), and household income (continuous variable). In addition, for the income of elderly parents, the logarithm is used for analysis to regress the unbiased results.

### Research Methods

STATA15.0 was used to analyze how children's achievements impact their elderly parents' health. First, a descriptive statistical analysis was used to describe the data distribution of the mediating and control variables. Second, a bivariate correlation analysis was performed for key variables to determine the significant relationship between these variables. Furthermore, we used the least squares method to analyze the relationship between children's achievements and their elderly parents' health. Finally, we adopted the multiple-step multiple mediator model proposed by Taylor et al. (33) and Hayes (34) to measure the mediating effect of living pattern, children' financial support to their parents, and social capital, and non-parameter percentile Bootstrap method of deviation correction was used to estimate the coefficient.

## Empirical Test

### Analysis of Results

Table 1 reports the basic status of the 2014 CLASS sample used in this study (N = 10,460), mainly including the number of samples, ranges, means, standard deviations, and frequencies. The respondents average self-rated health score was 3.02 (SD = 0.86, range: 1–5), physical health score was 28.70 (SD = 3.88, range: 10–30), and mental health score was 25.17 (SD = 3.88, range: 10–30). The average years of education completed by children is 11.10 years (SD = 3.34, range: 0–22), and the household income of children is about 4.80 yuan/year (SD = 1.63, range: 0–15.12). In terms of mediating variables, 46.09% of respondents lived with their children, and 53.91% did not live with their children. The annual financial aid provided by children is approximately 4.10 yuan/year (SD = 0.34, range: 0–5.12). The respondents' average social network score was about 20.18 points (SD = 8.62, range: 0–30). In terms of control variables, the respondents' average age was 69.49 years (SD = 7.52, range: 60–113). A total of 54.28% of respondents were male and 45.72% were female. Respondents who had a spouse (79.00%) had a much higher proportion than those with no spouse, single, divorced, or widowed (21.00%). The respondents' education levels were mainly junior high school and high school, with an average education completion of 9.29 years (SD = 5.34, range: 0–16). More than half of the respondents lived in urban areas (66.01%), and the remaining respondents lived in rural areas (33.99%). The average logarithm of the respondents' household income was 4.96 yuan/year (SD = 1.88, range: 0–11.51).

**Table 1:** Basic characteristics of variables.

**Variable**		**Obs**	**Range**	**Mean (SD)**	**Percent**
Dependent variable	Self-rated health (SRH)	10,460	1–5	3.02 (0.86)	
	Physical health (IADL)	10,460	10–30	28.70 (2.62)	
	Mental health (CESD)	10,460	10–30	25.17 (3.88)	
Independent variable	Children's education years	10,460	0–22	11.10 (3.34)	
	Children's income level	10,460	0–15.12	6.28 (1.63)	
Mediating variable	Housing arrangements	10,128	0–1		
	Living with children				46.09
	Not living with children				53.91
	Children' financial support	10,128	0–5.12	2.13 (0.34)	
	Social capital	10,128	0–30	20.18 (8.62)	
Control variable	Age	10,460	60–113	69.49 (7.52)	
	Gender	10,460	0–1		
	Female				45.72
	Male				54.28
	Marital status	10,460	0–1		
	With spouse				29.00
	Single, divorced or widowed				71.00
	Education years	10,460	0–16	9.29 (5.34)	
	Place of residence	10,460	0–1		
	Rural				33.99
	Urban				66.01
	Logarithm of family income	10,460	0–11.51	9.14 (1.88)	

### Bivariate Relationship Among Key Variables

We conducted a bivariate correlation analysis to test the associations among the key variables (see Table 2). First, self-rated health score (β = 0.387, *p* < 0.001), physical health score (β = 0.352, *p* < 0.001), mental health score (β = 0.185, *p* < 0.001), and children's income (β = 0.174, *p* < 0.001) were significantly related to children's education levels. Second, physical health was positively correlated with mental health (β = 0.292, *p* < 0.001), children's income (β = 0.118, *p* < 0.001), and children's education (β = 0.267, *p* < 0.001). Furthermore, mental health was positively correlated with children's income (β = 0.170, *p* < 0.001) and children's education (β = 0.222, *p* < 0.001). Finally, children's income was positively correlated with children's education (β = 0.295, *p* < 0.001).

**Table 2:** Correlation analysis between main variables.

**Variable name**	**Self-rated health**	**Physical health**	**Mental health**	**Children's income**	**Children's education**
Self-rated health	1				
Physical health	0.387^***^	1			
Mental health	0.352^***^	0.292^***^	1		
Children's income	0.185^***^	0.118^***^	0.170^***^	1	
Children's education level	0.174^***^	0.267^***^	0.222^***^	0.295^***^	1

*** *p < 0.01*.

### Empirical Tests

The regression results regarding the impact of children's achievements on their elderly parents' health are shown in Table 3. In Panel A, children's income [β = 0.240, 95%CI (0.208, 0.272), *p* < 0.001] significantly improved the self-rated health of their elderly parents. The higher the children's education levels [β = 0.015, 95%CI (0.007, 0.024), *p* < 0.001], the higher their elderly parents self-rated their health. In Panel B, the higher the children's income [β = 0.083, 95%CI (0.066, 0.099), *p* < 0.001], the higher the physical health scores of their elderly parents. Furthermore, children's education level [β = 0.009, 95%CI (0.004, 0.013), *p* < 0.001] had a significant positive correlation with the physical health scores of their elderly parents. That is, the higher the children's education levels, the higher the physical health scores of their elderly parents. In Panel C, both children's income [β = 0.359, 95%CI (0.290, 0.428), *p* < 0.001] and children's education [β = 0.056, 95%CI (0.038, 0.075), *p* < 0.001] can improve the mental health scores of their elderly parents. In other words, the higher the children's income and education levels, the lower the probability that their elderly parents will experience depression. This indicates that no matter which index is used to measure elderly parents' health, elderly parents' health status increases with the achievements of their children.

**Table 3:** Regression results about children's achievements and elderly health.

**Variable**	**β**	**SE**	**T-values**	***p*** **-values**	**95% CI**	
**Panel A**	**Self-rated health**					
Children's income	0.240	0.016	14.83	0.000	0.208	0.272
Children's education	0.015	0.004	3.67	0.000	0.007	0.024
Gender	0.100	0.024	4.10	0.000	0.052	0.148
Age	−0.015	0.002	−9.59	0.000	−0.018	−0.012
Marriage	−0.016	0.027	−0.60	0.548	−0.069	0.037
Education years	0.027	0.009	2.93	0.003	0.009	0.046
Place of residence	0.113	0.027	4.23	0.000	0.061	0.165
Logarithm of household income	0.035	0.006	5.64	0.000	0.023	0.048
**Panel B**	**Physical health**					
Children's income	0.083	0.008	9.99	0.000	0.066	0.099
Children's education	0.009	0.002	3.96	0.000	0.004	0.013
Gender	0.099	0.012	7.99	0.000	0.075	0.124
Age	−0.031	0.001	−38.72	0.000	−0.032	−0.029
Marriage	0.032	0.014	2.30	0.021	0.005	0.058
Education years	0.075	0.005	15.77	0.000	0.066	0.084
Place of residence	0.061	0.014	4.46	0.000	0.034	0.087
Logarithm of household income	0.029	0.003	9.05	0.000	0.023	0.035
**Panel C**	**Mental health**					
Children's income	0.359	0.035	10.23	0.000	0.290	0.428
Children's education	0.056	0.009	5.96	0.000	0.038	0.075
Gender	0.106	0.052	2.04	0.042	0.004	0.207
Age	−0.014	0.004	−3.90	0.000	−0.021	−0.007
Marriage	0.605	0.059	10.23	0.000	0.489	0.720
Education years	0.111	0.020	5.54	0.000	0.072	0.151
Place of residence	0.300	0.059	5.07	0.000	0.184	0.416
Logarithm of household income	0.066	0.015	4.48	0.000	0.037	0.095

The regression results show that control variables also have an important impact on elderly parents' health. Compared to female elderly parents, male elderly parents scored higher in self-rated health, physical health, and mental health. The older the elderly parents, the lower their self-rated health, physical health, and mental health scores. Compared with single elderly parents, married elderly parents scored higher in physical and mental health. The higher the education levels of elderly parents, the higher the scores on self-rated health, physical health, and mental health. Household income was significantly positively correlated with elderly parents' health. That is, the higher the family income of elderly parents, the higher their self-rated health, physical health, and mental health scores.

### The Mediation Effect of Housing Arrangements, Children' Financial Support and Social Capital

#### Living Patterns

Table 4 reports the mediating effect test on living patterns. In Model 1, children's education is significantly positive at the statistical level of 5%, indicating that there is a greater probability that elderly parents will live with their children as the children's education level increases. The children's income is significantly positive at the statistical level of 1%, indicating that there is a greater probability that elderly parents will live with their children as the children earn higher incomes. In Model 2, the elderly parents who live with their children have better self-rated health compared to elderly parents who do not live with their children. In Model 3, living pattern is significantly positive at the statistical level of 5%, indicating that elderly parents living with their children have better physical health. In Model 4, living pattern is significantly positive at the statistical level of 1%, and elderly parents living with their children have higher mental health scores. It is indicated that living pattern plays a mediating role in the relationship between children's achievements and their elderly parents' health. Hypothesis 2 is established.

**Table 4:** Analysis of multiple mediation effect of housing arrangement.

**Variable**	**Living pattern**	**Self-rated health**	**Physical health**	**Mental health**
	**(1)**	**(2)**	**(3)**	**(4)**
Children's education	0.012^**^	0.019^***^	0.010^***^	0.058^***^
	(0.006)	(0.005)	(0.002)	(0.010)
Children's income	0.110^***^	0.243^***^	0.083^***^	0.380^***^
	(0.022)	(0.018)	(0.009)	(0.037)
Living pattern		0.087^***^	0.033^**^	0.312^***^
		(0.027)	(0.014)	(0.055)
Control variables	Yes	Yes	Yes	Yes
Constant	2.835^***^	2.821^***^	2.883^***^	4.214^***^
	(0.189)	(0.150)	(0.077)	(0.326)
Observations	10,128	10,128	10,128	10,128

** *p < 0.05*,

*** *p < 0.01*.

#### Children' Financial Support to Their Parents

Table 5 reports the mediating effect test results related to children' financial support to their parents. In Model 1, children's education is significantly positively correlated with children' financial support to their parents. In other words, the higher the children's education level, the more children' financial support to their parents the elderly parents receive. Children's income is significantly positive at the statistical level of 1%, and elderly parents receive more children' financial support to their parents as their children's income increases. In Model 2, children' financial support to their parents is significantly positive at the statistical level of 1%. Elderly parents had higher self-rated health scores as they received more children' financial support to their parents. In Model 3, although the regression coefficient of children' financial support to their parents has a positive sign, it fails to pass the statistical level test. This indicates that children' financial support to their parents has no significant effect on the physical health of elderly parents. In Model 4, children' financial support to their parents has a positive effect on the mental health of elderly parents. That is, elderly parents have better mental health as they receive more children' financial support to their parents. Children' financial support to their parents plays a mediating role in the relationship between children's achievements and their elderly parents' health. Hypothesis 3 is established.

**Table 5:** Analysis of multiple mediation effect of children' financial support to their parents.

**Variable**	**Economic support**	**Self-rated health**	**Physical health**	**Mental health**
	**(1)**	**(2)**	**(3)**	**(4)**
Children's education	0.062^***^	0.020^***^	0.011^***^	0.046^***^
	(0.005)	(0.005)	(0.002)	(0.011)
Children's income	0.293^***^	0.245^***^	0.087^***^	0.334^***^
	(0.018)	(0.019)	(0.010)	(0.041)
Children' financial support to their parents		0.033^***^	0.003	0.101^***^
		(0.011)	(0.006)	(0.025)
Control variables	Yes	Yes	Yes	Yes
Constant	6.441^***^	3.172^***^	2.839^***^	3.786^***^
	(0.148)	(0.166)	(0.084)	(0.370)
Observations	10,128	10,128	10,128	10,128

*** *p < 0.01*.

#### Social Capital

Table 6 reports the mediating effect test results related to social capital. In Model 1, children's education is significantly positive at the statistical level of 1%. This indicates that the higher the children's education level, the richer the social capital of elderly parents. Children's income is significantly positively correlated with social capital. That is, the higher the children's income, the richer the social capital of their elderly parents. In Model 2, social capital is significantly positive at the 1% statistical level. This indicates that the richer the social capital, the higher the self-rated health score of elderly parents. In Model 3, social capital is significantly positively correlated with physical health. This indicates that the richer the social capital, the better the physical health of elderly parents. In Model 4, social capital has a positive effect on the mental health of elderly parents. That is, the richer the social capital, the better the mental health of elderly parents. Social network plays a mediating role in the relationship between children's achievements and their elderly parents' health. Hypothesis 3 is established.

**Table 6:** Analysis of multiple mediation effect of social capital.

**Variable**	**Social capital**	**Self-rated health**	**Physical health**	**Mental health**
	**(1)**	**(2)**	**(3)**	**(4)**
Children's education	0.153^***^	0.012^***^	0.008^***^	0.050^***^
	(0.025)	(0.004)	(0.002)	(0.009)
Children's income	0.890^***^	0.220^***^	0.069^***^	0.319^***^
	(0.096)	(0.016)	(0.008)	(0.035)
Social capital		0.022^***^	0.012^***^	0.050^***^
		(0.002)	(0.001)	(0.004)
Control variables	Yes	Yes	Yes	Yes
Constant	13.67^***^	2.573^***^	2.591^***^	3.694^***^
	(0.799)	(0.138)	(0.070)	(0.305)
Observations	10,128	10,128	10,128	10,128

*** *p < 0.01*.

## Conclusion and Discussion

Elderly health has significant economic and social consequences (15). As the population continues to age, a healthy aging strategy is particularly important in China's layout. In this context, although the responsibility for solving the predicament of aging has shifted from family to state, the traditional value of “filial piety” still plays an important role. As a result, healthy aging has become a collaboration among the country, society, and family. At the family level, children's achievements result in changes in family economic functions and lifestyle preferences, break the traditional structure of big families, and affect the health of elderly parents (3). Therefore, it is of great significance to investigate the impact of children's achievements on their elderly parents' health.

Regarding Chinese parents' cognition, children's achievements are mainly reflected in children's education and income levels. This study found that children's achievements (children's education and income) have a positive effect on the health of their elderly parents. Hypothesis 1 of this study was established. On the one hand, children with higher education levels have higher health literacy (such as reduced smoking and drinking and frequent physical examinations). Therefore, they reduce the risk of their parents' death related to chronic diseases, respiratory diseases, and lung cancer and improve the health of their elderly parents (35). At the same time, children's higher education levels can also delay the decline in their parents' cognitive function and short-term memory and maintain their parents' good mental state and perceived social status (30, 36). On the other hand, children with higher income levels will not only provide their parents with more elderly care services and improve their health, but they will also alleviate the mobility constraints faced by their elderly parents and improve the living standards of the elderly, enhance medical affordability, and strengthen their health (5). At the same time, Chinese people are sensitive about their reputation. If children have higher income, their elderly parents will feel dignified, which also plays a positive role in improving the mental health of the elderly (37).

This study finds that living patterns play a mediating role in the relationship between children's achievement and their elderly parents' health. Thus, Hypothesis 2 was established. With the advancement of new urbanization strategies and the continuous deepening of industrialization in China, young and middle-aged populations continue to migrate to urban areas. This results in the separation of adult children from their elderly parents and health declines in their elderly parents (38). However, high achieving children can relocate their families to be near them. Proximate residence, in which elderly parents live independently while children live nearby, is increasingly popular (9). Children living nearby can generally undertake more housework and visit more frequently than children who live far away (39), thus possibly improving and maintaining their elderly parents' health through timely supervision of the health behaviors and improved access to medical services (40, 41). In particular, children's concern about daily life and communication with the elderly (mental comfort) will help stimulate the elderly's intellectual development, thereby increasing the growth of brain nerves, maintaining low brain degeneration, and slowing down cognitive function decline (42).

This study finds that children' financial support to their parents plays a mediating role in the relationship between children's achievements and their elderly parents' health. Thus, Hypothesis 3 was established. Generally speaking, high achieving children maintain a higher socio-economic status and have a greater ability to provide material support to their elderly parents (43). Currently, China's old-age security system is incomplete and has a low security level. Traditional family pensions are still the main method of elderly care, and private transfer payments are an important income source for the elderly. Abundant evidence shows that transfer payments from children can significantly improve their parents' health (44, 45). Finch et al. (44) found that remittances from Mexican immigrants can significantly improve the accessibility of medical services for family members of origin. Gerber and Torosyan (45) use Georgia as an example and find that private transfer payments significantly increase the family budget and total family expenditure, which has a positive effect on the health of family members.

This study finds that social capital plays a mediating role in the relationship between children's achievements and their elderly parents' health. Thus, Hypothesis 4 was established. In China, a country that values human relationships, social capital plays an important role in people's daily lives. The intergenerational mobility model of Becker and Tomes (46) indicates that the maximization of family welfare needs to take intergenerational welfare level into account. There is a Chinese proverb that states, “We should see how the father loves the son in the first 30 years and how the son repays the father in the next 30 years.” This means that elderly parents have more social capital if their children achieve more. Social capital is strongly related to depression and chronic diseases, while trust in social capital can significantly alleviate depression symptoms in the elderly. Structure-based social capital can increase social participation of the elderly, encourage the elderly to participate in various organizational activities, enhance interpersonal communication, and reduce loneliness among the elderly (47–49). Furthermore, both community social capital and individual social capital promote the health of elderly individuals who live in rural areas. Interaction with friends, leisure, and entertainment, and helping others are the most important social activities that improve the health of elderly individuals who live in rural areas. Community social capital creates a greater health promotion effect than individual social capital (3).

This study has several important policy implications. First, based on the positive impact of children's achievements on parents' health, we need to vigorously promote the fine tradition of caring about children's education and create a strong atmosphere that fully supports education. On the other hand, with investment and resource allocation as the starting point, preferential policies should be released for children's education in disadvantaged families in terms of parental health. Secondly, in view of the effect of intergenerational connections in improving elderly parents' health, we need strengthen traditional cultural education of “filial piety” across the country, create a social atmosphere of “coming back home often,” and further broaden the applicability scope of paid leave system for employees who care for the elderly.

Of course, there are some limitations to this study. This study, with cross-sectional data from 2014, cannot identify the causal relationship between children's achievements and their elderly parents' health. At the same time, there may be a two-way causal relationship between children's achievements and their elderly parents' health, while two-way causality has been a major problem faced by social sciences for a long time. This study failed to find suitable instrumental variables to identify causal relationships. In addition, the three mediating variables of living pattern, children' financial support to their parents, and social capital interact in between, but this study regards them as parallel and does not analyze them based on multiple mediating effects.

## Data Availability Statement

The original contributions presented in the study are included in the article/supplementary material, further inquiries can be directed to the corresponding author/s.

## Author Contributions

YL and PZ designed the study. YL conducted the primary statistical analysis. Both authors contributed to the writing and revisions.

## Funding

This work was support by Ministry of Education of the People's Republic of China Humanities and Social Sciences Youth Foundation (Project No. 20YJCZH103).

## Conflict of Interest

The authors declare that the research was conducted in the absence of any commercial or financial relationships that could be construed as a potential conflict of interest.

## Publisher's Note

All claims expressed in this article are solely those of the authors and do not necessarily represent those of their affiliated organizations, or those of the publisher, the editors and the reviewers. Any product that may be evaluated in this article, or claim that may be made by its manufacturer, is not guaranteed or endorsed by the publisher.
